# Methanogenic degradation of lignin-derived monoaromatic compounds by microbial enrichments from rice paddy field soil

**DOI:** 10.1038/srep14295

**Published:** 2015-09-24

**Authors:** Souichiro Kato, Kanako Chino, Naofumi Kamimura, Eiji Masai, Isao Yumoto, Yoichi Kamagata

**Affiliations:** 1 Bioproduction Research Institute, National Institute of Advanced Industrial Science and Technology (AIST), 2-17-2-1 Tsukisamu-Higashi, Toyohira-ku, Sapporo, Hokkaido 062-8517, Japan; 2 Division of Applied Bioscience, Graduate School of Agriculture, Hokkaido University, Kita-9 Nishi-9, Kita-ku, Sapporo, Hokkaido 060-8589, Japan; 3 Research Center for Advanced Science and Technology, The University of Tokyo, 4-6-1 Komaba, Meguro-ku, Tokyo 153-8904, Japan; 4 Department of Bioengineering, Nagaoka University of Technology, 1603-1 Kamitomioka, Nagaoka, Niigata 940-2188, Japan

## Abstract

Anaerobic degradation of lignin-derived aromatics is an important metabolism for carbon and nutrient cycles in soil environments. Although there are some studies on degradation of lignin-derived aromatics by nitrate- and sulfate-reducing bacteria, knowledge on their degradation under methanogenic conditions are quite limited. In this study, methanogenic microbial communities were enriched from rice paddy field soil with lignin-derived methoxylated monoaromatics (vanillate and syringate) and their degradation intermediates (protocatechuate, catechol, and gallate) as the sole carbon and energy sources. Archaeal community analysis disclosed that both aceticlastic (*Methanosarcina* sp.) and hydrogenotrophic (*Methanoculleus* sp. and *Methanocella* sp.) methanogens dominated in all of the enrichments. Bacterial community analysis revealed the dominance of acetogenic bacteria (*Sporomusa* spp.) only in the enrichments on the methoxylated aromatics, suggesting that *Sporomusa* spp. initially convert vanillate and syringate into protocatechuate and gallate, respectively, with acetogenesis via *O*-demethylation. As the putative ring-cleavage microbes, bacteria within the phylum *Firmicutes* were dominantly detected from all of the enrichments, while the dominant phylotypes were not identical between enrichments on vanillate/protocatechuate/catechol (family *Peptococcaceae* bacteria) and on syringate/gallate (family *Ruminococcaceae* bacteria). This study demonstrates the importance of cooperation among acetogens, ring-cleaving fermenters/syntrophs and aceticlastic/hydrogenotrophic methanogens for degradation of lignin-derived aromatics under methanogenic conditions.

Lignin is a major component of terrestrial plants and is a highly complex heteropolymer consisting of hydoxylated and methoxylated phenylpropanid units linked by various types of C–C and C–O-C bonds. As lignin represents a significant part of input of organic compounds, its degradation is integral for carbon and energy cycles in soil environments. Lignin is aerobically depolymerized by lignolytic fungi and some bacterial strains, followed by mineralization of the resulting low-molecular-weight products by soil bacteria^1^,^2^,^3^. Aerobic degradation of lignin-derived methoxylated monoaromatics, including vanillate (4-hydroxy-3-methoxybenzoate) and syringate (3,5-dimethoxy-4-hydroxybenzoate), has been thoroughly studied^4^. For example, an aerobic vanillate- and syringate-degrading bacterium *Sphingobium* sp. SYK-6 firstly convert vanillate and syringate into protocatechuate (PCA, 3,4-dihydroxybenzoate) and 3-*O*-methylgallate (3-MGA), respectively, by *O*-demethylation reactions^5^. PCA is oxidatively decomposed to pyruvate and oxaloacetate via the PCA 4,5-cleavage pathway^6^. 3-MGA is degraded through multiple ring cleavage pathways and the resultant metabolites are further degraded via the PCA 4,5-cleavage pathway^7^.

On the contrary, studies on anaerobic degradation of lignin and lignin-derived aromatics have been limited, despite some studies suggesting that their anaerobic biodegradation was evident in various natural environments^8^,^9^,^10^,^11^,^12^,^13^. Acetogenic bacteria are the first anaerobes described to utilize methoxylated aromatics as the sole carbon and energy sources^14^,^15^. Acetogenic bacteria utilize the *O*-methyl group as the carbon and energy sources, while they do not have the ability to degrade the aromatic ring structure. Similarly, some sulfate-reducing bacteria and fermentative bacteria utilize the *O*-methyl group as carbon and energy sources, and a part of them also have ability to decompose the aromatic rings^16^,^17^,^18^,^19^. Anaerobic degradation of the *O*-demethylated derivatives of vanillate and syringate, namely PCA and gallate (3,4,5-trihydroxybenzoate), respectively, has been documented with nitrate-reducing, sulfate-reducing, and fermentative bacteria. A nitrate-reducer *Thauera aromatica* and a sulfate-reducer *Desulfobacterium* sp. were reported to anaerobically degrade PCA via the benzoyl-CoA pathway^20^,^21^. Fermentative bacteria, such as *Eubacterium oxidoreducens* and *Pelobacter acidigallici*, were reported to anaerobically degrade gallate via the phloroglucinol pathway^22^,^23^.

While methanogenesis is one of the most important microbial metabolisms in diverse anaerobic environments, including soil and freshwater/marine sediments, knowledge on methanogenic degradation of lignin-derived aromatics, especially on microorganisms responsible for the decomposition, has been quite limited. Kaiser and Hanselmann reported that microbial communities enriched from anaerobic sediments completely degraded lignin-derived aromatics, including vanillate and syringate, with concomitant generation of CH_4_ (ref. 24). They demonstrated that the first step for methanogenic degradation is acetate production with *O*-demethylation of the methoxy-group. However, microorganisms participating in the *O*-demethylation and following ring-cleavage reactions under methanogenic environments were not identified.

In the present study, we enriched methanogenic microbial communities from rice paddy field soil with either lignin-derived aromatics (vanillate and syringate) or a model aromatic compound (benzoate) as the sole carbon and energy sources. The microbial communities were further enriched on the degradation intermediate compounds, namely PCA, catechol, and gallate. Microorganisms involved in methanogenic degradation of the lignin-derived aromatics were then identified with microbial community analysis based on their 16S rRNA gene sequences.

## Results and Discussion

### Enrichment of methanogenic microbial communities on lignin-derived aromatics

**Figure 1:**
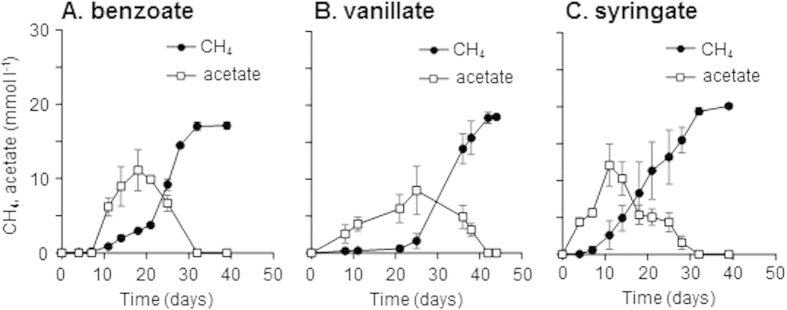
Methanogenesis from benzoate, vanillate, and syringate by enrichment cultures. Production of CH_4_ (filled circles) and acetate (open squares) were monitored during the incubation of respective enrichment cultures supplemented with 5 mM of benzoate (**A**), vanillate (**B**), or syringate (**C**). Data are presented as the means of three independent cultures, and error bars represent standard deviations.

Methanogenic microbial communities were enriched from rice paddy field soil using a freshwater basal medium supplemented with different aromatic compounds. Either lignin-derived methoxylated aromatics (5 mM of vanillate or syringate) or a control aromatic compound (5 mM of benzoate) were supplemented as the substrates. After several weeks of cultivation, CH_4_ was produced in all of the cultures supplemented with aromatics (Fig. S1). The amount of CH_4_ produced reached to 7 to 20 mmol l^−1^ within 2 month cultivation. On the contrary, only trace amount of CH_4_ (less than 0.1 mmol l^−1^) was produced in the cultures without supplementation of the aromatics (Fig. S1), indicating that most of CH_4_ produced in the enrichment cultures was derived from degradation of aromatic compounds. Production of CH_4_ from the aromatic compounds by respective enrichments (after five successive subcultures) are shown in Fig. 1A–C. It took more than one month to produce stoichiometrically expected CH_4_ in all cultures tested. The amounts of CH_4_ produced from 5 mM of benzoate, vanillate, and syringate were 17.1 ± 0.5, 18.4 ± 0.3, and 20.1 ± 0.3 mmol l^−1^, respectively, and were approximate to the theoretical values (18.75, 20, and 22.5 mmol l^−1^, respectively) calculated from the following equations:


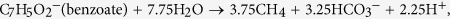



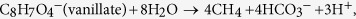



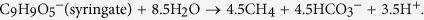


These results suggest that the each enrichment culture completely degraded the aromatic compounds into CH_4_ and CO_2_.

### Detection of degradation intermediates from the enrichment cultures

Among short chain alcohols and organic acids, only acetate was detected from the enrichment cultures throughout this study by the high performance liquid chromatography (HPLC) analysis. Acetate accumulated in the enrichment cultures to 8 to 12 mM during methanogenesis, followed by the complete consumption of the accumulated acetate (Fig. 1A–C), indicating that acetate is one of the important intermediates of methanogenic degradation of the aromatic compounds.

**Figure 2:**
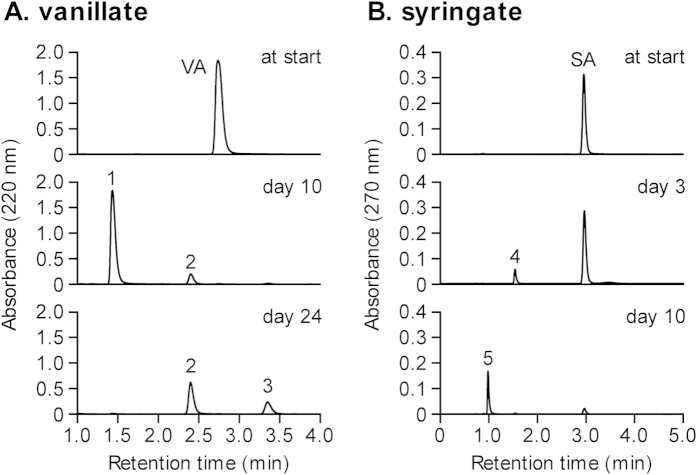
Degradation of vanillate and syringate by enrichment cultures. The cultures were incubated with 5 mM of vanillate (**A**) or syringate (**B**), and portions of the enrichments were corrected at the start, day 10, and day 24 or at the start, day 3, and day 10, respectively. The supernatant of enrichment of vanillate and syringate were analyzed by HPLC with detection at 220 nm and 270 nm, respectively. The retention times of compound I, II, III, IV, and V were 1.43, 2.40, 3.34, 1.53, and 0.98 min, respectively. UV/VIS and mass spectra of compound I to V are shown in Figs S2 and S3.

In order to determine the degradation pathway of vanillate and syringate in the enrichment cultures, intermediate metabolites produced during cultivation were analyzed by HPLC coupled with an electrospray ionization-mass spectrometry (ESI-MS). It should be noted that CoA-derivatives of aromatic compounds could not be detected by our analytical protocol. In the vanillate enrichment cultures, two peaks (compound I, II) appeared at day 10, followed by an appearance of another peak (compound III) at day 24 (Fig. 2A). Based on the comparison of retention time, UV/VIS spectrum, and negative ESI-MS spectrum with authentic compounds, compound I, II, and III were identified as PCA, catechol, and 3-hydroxybenzoate (3-HB), respectively (Figs S2 and S3). Generation of PCA at day 10 suggests that *O*-demethylation of vanillate is the first step of vanillate degradation. Catechol and 3-HB were appeared to be produced by the decaroboxylation and dehydroxylation of PCA, respectively, probably as intermediates of methanogenic degradation pathway of PCA (discussed below). In the degradation of syringate, two metabolites, compound IV and V, were found and identified as 3-MGA and gallate, respectively (Fig. 2B, S2, and S3). These observations indicate that syringate is first converted to gallate with successive *O*-demethylation reactions via 3-MGA. In the benzoate enrichment cultures, no aromatic compound other than benzoate were detected (data not shown), suggesting that benzoate is firstly converted to benzoyl-CoA and then the ring structure is reductively cleaved via benzoyl-CoA pathway as reported^25^,^26^.

### Enrichment cultures on the degradation intermediates

**Figure 3:**
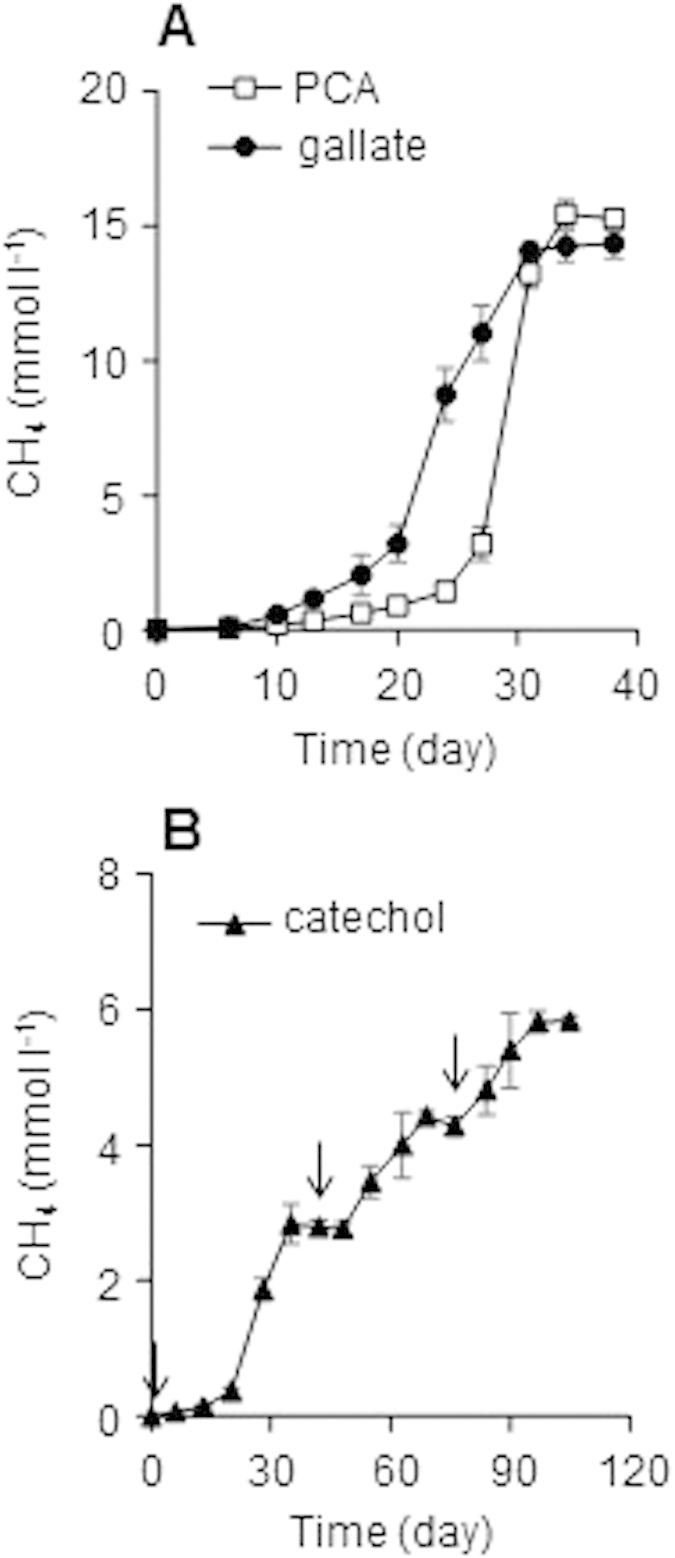
Methanogenesis from PCA, gallate, and catechol by respective enrichment cultures. PCA (open squares, (**A**)) and gallate (filled circles, (**A**)) were supplemented with 5 mM at day 0. Catechol (filled triangles, (**B**)) was supplemented with 1  M at the time points represented by arrows. Data are presented as the means of three independent cultures, and error bars represent standard deviations.

In order to further elucidate the mechanisms for degradation of the lignin-derived aromatics, the enrichment cultures on vanillate and syringate were subjected to further enrichments with respective degradation intermediates. Enrichment cultures on PCA and gallate were constructed with vanillate- and syringate-enrichments as the inoculum, respectively, and generated considerable amounts of CH_4_ within 40 days (Fig. 3A). Although enrichment cultures on catechol was also constructed using the vanillate-enrichments as the inoculum, the cultures supplemented with 5 mM catechol produced only little amounts of CH_4_ with a long lag time (>50 days, data not shown). Since cytotoxicity of relatively high concentration of catechol was considered as the reason for the low methanogenic activity, another set of enrichment cultures was constructed with supplementation of 1 mM catechol. In this case, methanogenesis started with approximately a 20-day lag phase and plateaued at around 40 days of cultivation (Fig. 3B). After saturation of methanogenesis, another 1 mM of catechol was added to the culture, which resulted in resumption of methanogenesis with almost no lag time.

The amount of CH_4_ produced from PCA (5 mM), gallate (5 mM), and catechol (1 mM) were 15.5 ± 0.5, 14.4 ± 0.5, and 2.8 ± 0.3 mmol l^−1^, respectively, and were approximate to the theoretical values (16.25, 15, and 3.25 mmol l^−1^, respectively) calculated from the following equations:


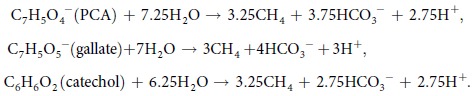


These results suggest that methanogenic microbial communities that completely degrade the respective intermediates of vanillate and syringate were successfully enriched.

### Dominant microorganisms in the enrichment cultures

**Figure 4:**
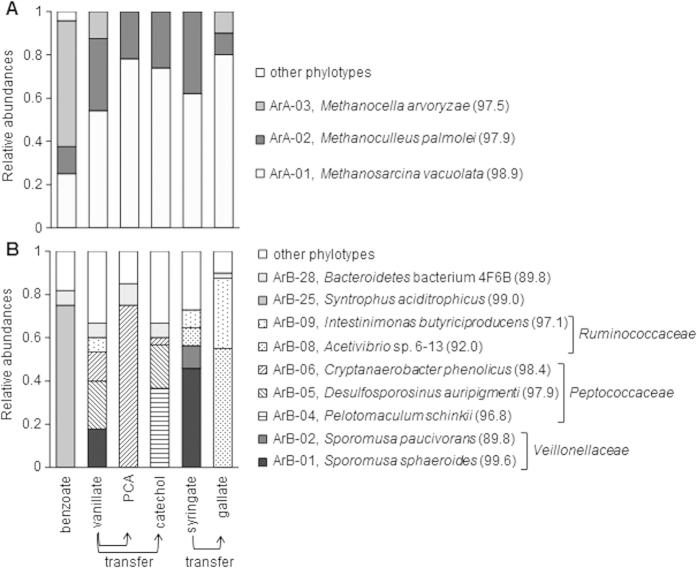
Phylogenetic distribution of archaeal (A) and bacterial (B) 16S rRNA gene clones in the aromatics-degrading methanogenic enrichment cultures. The dominant phylotypes (>10% in at least one enrichment) and their closest relatives (sequence identity, %) are shown in the legends.

Microbial community structures of the enrichments were assessed based on 16S rRNA gene clone library analysis to elucidate the microorganisms participating in methanogenic degradation of the lignin-derived aromatics. The summarized features of the bacterial and archaeal community analyses are presented in Fig. 4. All phylotypes detected from each enrichment culture are listed in Tables S1 and S2.

Only 4 archaeal phylotypes were recovered from the enrichment cultures (Fig. 4A and Table S1). The phylotype ArA-01 (98.9% identity to *Methanosarcina vacuolata*), which dominated in all enrichment cultures, was the only phylotype classified as an aceticlastic methanogen. The phylotypes classified as hydrogenotrophic methanogens, namely the phylotype ArA-02 (97.9% identity to *Methanoculleus palmolei*) and ArA-03 (97.5% identity to *Methanocella arvoryzae*), were also detected from all enrichment cultures. These results suggest that both aceticlastic and hydrogenotrophic methanogenesis contribute to methanogenic degradation of the lignin-derived aromatics.

The predominant bacterial phylotype in the benzoate-enrichments was the phylotype ArB-25 (Fig. 4B and Table S2), which is classified into the class *Deltaproteobacteria* and has 99.0% identity to *Syntrophus aciditrophicus*. *S. aciditrophicus* has ability to degrade benzoate via benzoyl-CoA pathway in syntrophic association with methanogens^27^,^28^. In the benzoate-enrichment cultures, *Syntrophus* sp. appears to decompose benzoate into acetate and H_2_/CO_2_, which is further converted into CH_4_ by aceticlastic and hydrogenotrophic methanogens.

By contrast, almost no *Deltaproteobacteria* were recovered from the enrichment cultures with other aromatic compounds. Instead, the phylotypes classified into phylum *Firmicutes* dominated in the enrichments supplemented with the lignin-derived aromatics and their degradation intermediates (Fig. 4B). Among the *Firmicutes* phylotypes, the phylotype ArB-01 (99.6% identity to *Sporomusa sphaeroides*) and ArB-02 (89.8% identity to *Sporomusa paucivorans*) were abundantly detected from vanillate- and syringate-enrichments, but not from enrichments with their degradation intermediates. *Sporomusa* spp. were reported to have the ability to acetogenetically grow on diverse array of methoxylated aromatic compounds, such as vanillate, ferulate, and 3,4-dimethoxybenzoate, via respective *O*-demethylation reactions^29^,^30^. These findings suggested that *Sporomusa* spp. contribute to the initial step of anaerobic degradation of the lignin-derived aromatics in the enrichments, namely the conversion of vanillate and syringate into PCA and gallate, respectively.

**Figure 5:**
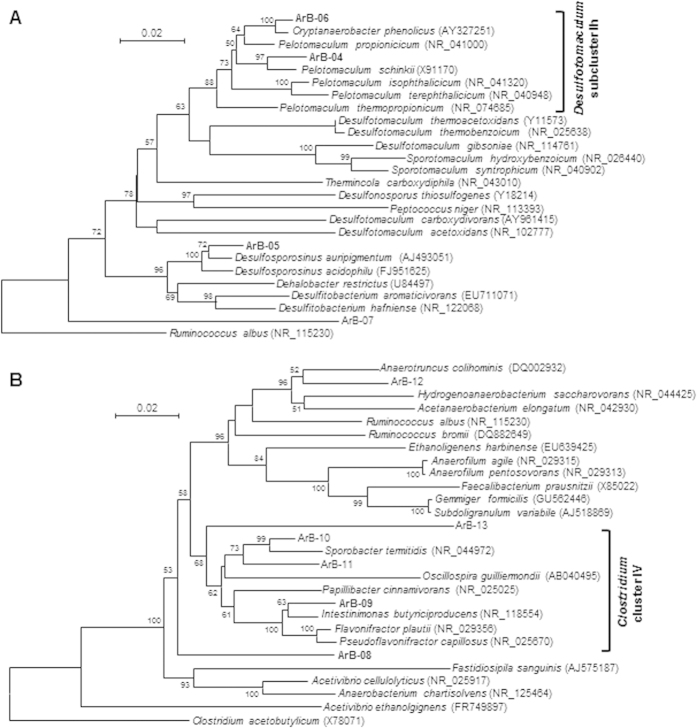
A phylogenetic tree based on partial 16S rRNA gene sequences of the phylotypes retrieved in this study and representative isolates in family *Peptococcaceae* (A) and *Ruminococcaceae* (B). The phylotypes dominantly detected from the enrichment cultures are indicated by bold letters*. Ruminococcus albus* (**A**) and *Clostridium acetobutylicum* (**B**) were used as the outgroup sequences. Accession numbers are shown in parentheses. Bootstrap values (1000 trials, only > 50% are shown) are indicated at branching points. The bar indicates 2% sequence divergence.

In the enrichments on vanillate and its degradation intermediates (PCA and catechol), the phylotypes classified into family *Peptococcaceae* were dominant, while the dominant phylotypes varied among the supplemented aromatic compounds (Fig. 4B and Table S2). The phylogenetic tree based on 16S rRNA gene sequences of representative *Peptococcaceae* isolates and the *Peptococcaceae* phylotypes detected in this study is shown in Fig. 5A. The dominant phylotypes ArB-04 (96.8% identity to *Pelotomaculum schinkii*) and ArB-06 (98.4% identity to *Cryptanaerobacter phenolicus*) were clustered into the *Desulfotomaculum* subcluster Ih^31^. Microorganisms in the *Desulfotomaculum* subcluster Ih were characterized by their ability to anaerobically metabolize various organic substrates in syntrophic association with hydrogenotrophic methanogens^32^. Furthermore, *Desulfotomaculum* subcluster Ih includes some strains that syntrophically degrade a range of aromatic compounds^33^,^34^ and was frequently detected as the dominant bacteria in microbial communities that anaerobically degrade diverse aromatic compounds^35^,^36^,^37^,^38^. The other dominant phylotype ArB-05 (97.9% identity to *Desulfosporosinus auripigmenti*) was separately clustered from ArB-04 and -06 and affiliated with genus *Desulfosporosinus* (Fig. 5A). *Desulfosporosinus* spp. were generally characterized as sulfate-reducing bacteria and its syntrophic metabolism with methanogens has not been tested^39^. However, all crucial domains for syntrophic metabolisms were found in the genome of *Desulfosporosinus meridiei*, suggesting their possible ability to grow in syntrophic association with methanogens^40^. While there have been no reports on degradation of aromatic compounds by *Desulfosporosinus* spp., sequences related to *Desulfosporosinus* were frequently recovered from various aromatics-degrading microbial communities^41^,^42^,^43^. Taken together, it is very likely that the *Peptococcaceae* phylotypes mainly contributed to the ring-cleavage of the vanillate and/or its degradation intermediates via syntrophic interaction with hydrogenotrophic methanogens.

The phylotypes classified into family *Ruminococcaceae* were dominant in the enrichments with syringate and gallate (Fig. 4B and Table S2). The phylogenetic tree for family *Ruminococcaceae* is shown in Fig. 5B. One of the phylotypes, ArB-08, was not clustered with known *Ruminococcaceae* isolates and has only quite low 16S rRNA gene sequence identity to isolated microorganisms (92.0% identity to *Acetivibrio* sp. 6–13). The other dominant phylotype ArB-09 (97.1% identity to *Intestinimonas butyriciproducens*) was placed within the “*Clostridium* cluster IV^44^,^45^”. Although the closest relative of ArB-09 (*I. butyriciproducens*) has not been reported to degrade aromatic compounds^46^, some strains in the *Clostridium* cluster IV were reported to fermentatively decompose diverse aromatic compounds. For example, *Flavonifractor plautii* (formerly *Clostridium orbiscindens*) has the ability to degrade flavonoids via phloroglucinol (1,3,5-trihydroxybenzene) as the intermediate^47^. Most notably, *Sporobacter termitidis* grows exclusively on a limited range of methoxylated aromatic compounds, including syringate and vanillate, through their *O*-demethylation and ring cleavage^17^. These reports support the assumption that the *Ruminococcaceae* phylotypes dominant in the syringate- and gallate-enrichments mainly contributed to their ring cleavage.

### Proposed model for methanogenic degradation of vanillate and syringate

**Figure 6:**
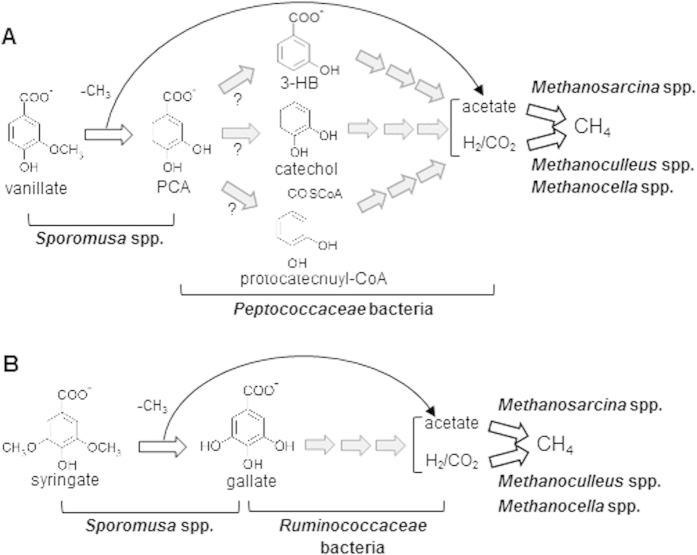
The proposed models for methanogenic degradation of vanillate (A) and syringate (B) in the enrichment cultures. Microorganisms expected to catalyze each reaction are presented in bold letters. Gray arrows represent reactions with unidentified pathway(s).

The proposed models for methanogenic degradation of the lignin-derived aromatics are shown in Fig. 6. Analysis on degradation intermediates and microbial communities in the enrichments suggested that vanillate is firstly converted into PCA by *Sporomusa* spp. with acetate generation via *O*-demethylation reactions (Fig. 6A). *Peptococcaceae* bacteria (*Desulfotomaculum* subcluster Ih and/or *Desulfosporosinus* spp.) are the plausible candidates for decomposing PCA into acetate and H_2_/CO_2_, which are the substrates for methanogenesis by aceticlastic methanogens (*Methanosarcina* spp.) and hydrogenotrophic methanogens (*Methanoculleus* and *Methanocella* spp.), respectively. Anaerobic degradation of PCA has been investigated for some nitrate- and sulfate-reducing bacteria^20^,^21^, by which PCA is firstly activated to protocatechuyl-CoA and reductively dehydroxylated to 3-HB-CoA, followed by ring cleavage via the (3-hydroxy)benzoyl-CoA pathway. However, we could not confirm the existence of this degradation pathway in the enrichment cultures, because CoA-derivatives of aromatic compounds could not be detected by our analytical protocol. Instead, catechol and 3-HB were detected as the possible intermediate compounds through degradation of PCA (Fig. 2A). Although there have been no reports on microbial enzymes that dehydroxylate PCA into 3-HB under anoxic conditions, it might be possible that certain bacteria utilize such reaction followed by CoA activation and ring cleavage via (3-hydroxy)benzoyl-CoA pathway. While enzymatic activities to generate catechol by decarboxylation of PCA were reported for some anaerobic microorganisms^48^,^49^, further catechol degradation routes have not been identified. Some nitrate- and sulfate-reducing bacteria were reported to decompose catechol^20^,^50^. These bacteria initially convert catechol into PCA by carboxylation, followed by activation with CoA-ligation, dehydroxylation and reductive ring cleavage via the (3-hydroxy)benzoyl-CoA pathway. Although there are some reports on anaerobic degradation of catechol under methanogenic conditions^51^,^52^,^53^, the responsible microorganisms and the degradation pathway were not identified.

As in the case of vanillate, the first step of syringate degradation appears to be acetogenic *O*-demethylation catalyzed by *Sporomusa* spp. (Fig. 6B). The demethoxylated product, gallate, seems to be further decomposed into acetate and H_2_/CO_2_ mainly by *Ruminococcaceae* bacteria. Anaerobic degradation of gallate were reported for some fermenting bacteria, including *E. oxidoreducens*, which is classified into *Firmicutes* and distantly related to the *Ruminococcaceae* phylotypes detected in this study^22^,^23^. *E. oxidoreducens* initially converts gallate into trihydroxybenzene by decarboxylation reaction, followed by ring cleavage via the phloroglucinol pathway^22^. Although the decarboxylated products of gallate were not detected in this study, it is highly possible that the trihydroxybenzene degradation is much faster than its generation.

### Concluding remarks

In this study, we successfully enriched methanogenic microbial communities that decompose the lignin-derived monoaromatics, namely syringate and vanillate. This study is the first to demonstrate that cooperation of microorganisms with a diverse range of trophic groups are required for methanogenic degradation of the lignin-derived aromatics. The initial step appears to be catalyzed by *Sporomusa* spp. that generate acetate via *O*-demethylation of the methoxylated aromatics. The resultant demethoxylated aromatics were decomposed into acetate and H_2_/CO_2_ by *Firmicutes* bacteria, while the bacterial groups responsible for PCA (family *Peptococcaceae*) and gallate (family *Ruminococcaceae*) were not identical. Finally, both aceticlastic and hydrogenotrophic methanogens generate methane from acetate and H_2_/CO_2_, respectively. Although the details in the pathway of aromatic ring cleavage have not been characterized, isolation of aromatics-degrading *Peptococcaceae* and *Ruminococcaceae* strains identified in this study followed by genomic and enzymatic studies will shed light on novel aspects of methanogenic degradation of lignin-derived aromatics in anaerobic environments.

## Methods

### Enrichment cultures of methanogenic microbial communities

Methanogenic microbial communities were enriched in serum bottles (68 ml in capacity) filled with 20 ml of a bicarbonate- and HEPES-buffered freshwater basal medium (pH 7.0)^54^ supplemented with aromatic compounds (benzoate, vanillate, or syringate) as a substrate. The cultures without supplementation of organic substrates were also prepared as a no-amendment control. Approximately 50 mg (wet weight) of rice paddy field soil was inoculated as a microbial source. The cultures were incubated at 30 °C under an atmosphere of N_2_/CO_2_ [80:20 (v/v)] without shaking. When methanogenesis reached a plateau, 0.5 ml of culture solution was transferred to the fresh media. Enrichment cultures on PCA/catechol and gallate were constructed in the same medium with the vanillate- and syringate-enrichments as the inoculum, respectively. After more than three passages, the enrichment cultures were subjected to chemical and phylogenetic analyses. All culture experiments were conducted in triplicate.

### Chemical analyses

The partial pressure of CH_4_ was determined using a gas chromatograph (GC-2014, Shimadzu) as described previously^55^. The concentrations of organic acids were determined using a HPLC (D-2000 LaChrom Elite HPLC system, HITACHI) as described previously^56^. To identify metabolic intermediates, the authentic compounds and supernatant of enrichment cultures were analyzed by HPLC (ACQUITY UPLC system, Waters) coupled with ESI-MS (ACQUITY TQ detector, Waters) using a TSKgel ODS-140HTP column (2.1 by 100 mm, Tosoh) as described previously^57^. In the HPLC analysis, the mobile phase was a 90:10 (v/v) water:acetonitrile at a flow rate of 0.3 ml/min. Formic acid (0.1%) was added to the mobile phase solvent as a means of increasing ionization efficiency for ESI-MS. Degradation products of vanillate and syringate were detected at the wavelength of 220 nm and 270 nm, respectively. Wavelength for the detection and the retention times of authentic compounds were described in Figs S2 and S3. In the ESI-MS analysis, mass spectra were obtained by negative and positive modes with the following settings: capillary voltage, 3.0 kv; cone voltage, 10 to 40 V; source temperature, 120 °C; desolvation temperature, 350 °C; desolvation gas flow rate, 650 liter h^−1^; and cone gas flow rate, 50 liter h^−1^.

### Phylogenetic analyses

Microbial DNA was extracted with the FAST DNA Spin Kit for soil (MP Biomedicals) according to the manufacturer’s instructions. PCR amplification of 16S rRNA gene fragments for clone library analyses was conducted as described previously^58^, with primer sets of U515f and U1492r for bacteria and A25f and A958r for archaea^59^. The sequences obtained were assigned to each phylotype using BLASTClust program^60^ with a cut-off value of 97% sequence identity. Classification of phylotypes was performed using the Classifier program in the Ribosomal Database Project database^61^. The sequence of each phylotype was compared to those stored in the GenBank nucleotide sequence database using the BLAST program^60^ to infer the closest relatives. Phylogenetic trees were constructed by the neighbor-joining method^62^ using program MEGA^63^. The bootstrap resampling method^64^ was used with 1000 replicates to evaluate the robustness of the phylogenetic trees.

### Nucleotide sequence accession numbers

The nucleotide sequence data reported here have been submitted to GenBank under Accession No. LC036665–LC036702.

## Additional Information

**How to cite this article**: Kato, S. *et al.* Methanogenic degradation of lignin-derived monoaromatic compounds by microbial enrichments from rice paddy field soil. *Sci. Rep.* **5**, 14295; doi: 10.1038/srep14295 (2015).

## Supplementary Material

Supplementary Information

## References

[b1] Zimmermann W. Degradation of lignin by bacteria. J. Bacteriol. 13, 119–130 (1990).

[b2] Martínez A. T. *et al.* Biodegradation of lignocellulosics: microbial, chemical, and enzymatic aspects of the fungal attack of lignin. Int. Microbiol. 8, 195–204 (2005). 16200498

[b3] Bugg T. D., Ahmad M., Hardiman E. M. & Rahmanpour R. Pathways for degradation of lignin in bacteria and fungi. Nat. Prod. Rep. 28, 1883–1896 (2011). 21918777 10.1039/c1np00042j

[b4] Masai E., Katayama Y. & Fukuda M. Genetic and biochemical investigations on bacterial catabolic pathways for lignin-derived aromatic compounds. Biosci. Biotechnol. Biochem. 71, 1–15 (2007). 17213657 10.1271/bbb.60437

[b5] Abe T., Masai E., Miyauchi K., Katayama Y. & Fukuda M. A tetrahydrofolate-dependent O-demethylase, LigM, is crucial for catabolism of vanillate and syringate in *Sphingomonas paucimobilis* SYK-6. J. Bacteriol. 187, 2030–2037 (2005). 15743951 10.1128/JB.187.6.2030-2037.2005 PMC1064056

[b6] Kasai D., Masai E., Katayama Y. & Fukuda M. Degradation of 3-O-methylgallate in *Sphingomonas paucimobilis* SYK-6 by pathways involving protocatechuate 4,5-dioxygenase. FEMS Microbiol. Lett. 274, 323–328 (2007). 17645527 10.1111/j.1574-6968.2007.00855.x

[b7] Kamimura N. & Masai E. The protocatechuate 4,5-cleavage pathway: Overview and new findings. In Biodegradative Bacteria. Nojiri H., Tsuda M., Fukuda M. & Kamagata Y. (eds). Springer: Japan, , pp. 207–226 (2014).

[b8] Hackett W. F., Connors W. J., Kirk T. K. & Zeikus J. G. Microbial decomposition of synthetic ^14^C-labeled lignins in nature: lignin biodegradation in a variety of natural materials. Appl. Environ. Microbiol. 33, 43–51 (1977). 16345189 10.1128/aem.33.1.43-51.1977 PMC170572

[b9] Benner R., Maccubbin A. E. & Hodson R. E. Anaerobic biodegradation of the lignin and polysaccharide components of lignocellulose and synthetic lignin by sediment microflora. Appl. Environ. Microbiol. 47, 998–1004 (1984). 16346554 10.1128/aem.47.5.998-1004.1984 PMC240039

[b10] Young L. Y. & Frazer A. C. The fate of lignin and lignin-derived compounds in anaerobic environments. Geomicrobiol. J. 5, 261–293 (1987).

[b11] Cookson L. J. The site and mechanism of ^14^C-lignin degradation by *Nasutitermes exitiosus*. J. Insect. Physiol. 34, 409–414 (1988).

[b12] Dittmar T. & Lara R. Molecular evidence for lignin degradation in sulfate-reducing mangrove sediments (Amazonia, Brazil). Geochim. Cosmochim. Acta 65, 1417–1428 (2001).

[b13] Ko J. J. *et al.* Biodegradation of high molecular weight lignin under sulfate reducing conditions: lignin degradability and degradation by-products. Bioresource Technol. 100, 1622–1627 (2009). 10.1016/j.biortech.2008.09.029 18977138

[b14] Bache R. & Pfennig N. Selective isolation of *Acetobacterium woodii* on methoxylated aromatic acids and determination of growth yields. Arch. Microbiol. 130, 255–261 (1981).

[b15] Daniel S. L., Keith E. S., Yang H., Lin Y. S. & Drake H. L. Utilization of methoxylated aromatic compounds by the acetogen *Clostridium thermoaceticum*: expression and specificity of the co-dependent O-demethylating activity. Biochem. Biophys. Res. Commun. 180, 416–422 (1991). 1930235 10.1016/s0006-291x(05)81309-9

[b16] Tasaki M., Kamagata Y., Nakamura K. & Mikami E. Utilization of methoxylated benzoates and formation of intermediates by *Desulfotomaculum thermobenzoicum* in the presence or absence of sulfate. Arch. Microbiol. 157, 209–212 (1992). 1510551 10.1007/BF00245151

[b17] Grech-Mora I. *et al.* Isolation and characterization of *Sporobacter termitidis* gen. nov., sp. nov., from the digestive tract of the wood-feeding termite *Nasutitermes lujae*. Int. J. Syst. Bacteriol. 46, 512–518 (1996).

[b18] Mechichi T., Labat M., Garcia J., Thomas P. & Pate B. K. C. *Sporobacterium olearium* gen. nov., a new methanethiol-producing bacterium that degrades aromatic compounds, isolated from an olive mill wastewater treatment digester. Int. J. Syst. Bacteriol. 49, 1741–1748 (1999). 10555356 10.1099/00207713-49-4-1741

[b19] Neumann A. *et al.* Phenyl methyl ethers: novel electron donors for respiratory growth of *Desulfitobacterium hafniense* and *Desulfitobacterium* sp. strain PCE-S. Arch. Microbiol. 181, 245–249 (2004). 14758469 10.1007/s00203-004-0651-y

[b20] Gorny N. & Schink B. Anaerobic degradation of catechol by *Desulfobacterium* sp. strain Cat2 proceeds via carboxylation to protocatechuate. Appl. Environ. Microbiol. 60, 3396–3400 (1994). 7944370 10.1128/aem.60.9.3396-3400.1994 PMC201815

[b21] Philipp B. *et al.* Anaerobic degradation of protocatechuate (3,4-dihydroxybenzoate) by *Thauera aromatica* strain AR-1. FEMS Microbiol. Lett. 212, 139–143 (2002). 12076800 10.1111/j.1574-6968.2002.tb11257.x

[b22] Krumholz L. R. & Bryant M. P. Characterization of the pyrogallol-phloroglucinol isomerase of *Eubacterium oxidoreducens*. J. Bacteriol. 170, 2472–2479 (1988). 3372475 10.1128/jb.170.6.2472-2479.1988 PMC211158

[b23] Brune A. & Schink B. Phloroglucinol pathway in the strictly anaerobic *Pelobacter acidigallici*: fermentation of trihydroxybenzenes to acetate via triacetic acid. Arch. Microbiol. 157, 417–424 (1992).

[b24] Kaiser J. & Hanselmann K. Fermentative metabolism of substituted monoaromatic compounds by a bacterial community from anaerobic sediments. Arch. Microbiol. 133, 185–194 (1982).

[b25] Peters F., Shinoda Y., McInerney M. J. & Boll M. Cyclohexa-1,5-diene-1-carbonyl-coenzyme A (CoA) hydratases of *Geobacter metallireducens* and *Syntrophus aciditrophicus*: Evidence for a common benzoyl-CoA degradation pathway in facultative and strict anaerobes. J. Bacteriol. 189, 1055–1060 (2007). 17122342 10.1128/JB.01467-06 PMC1797300

[b26] Fuchs G., Boll M. & Heider J. Microbial degradation of aromatic compounds - from one strategy to four. Nat. Rev. Microbiol. 9, 803–816 (2011). 21963803 10.1038/nrmicro2652

[b27] Jackson B. & Bhupathiraju V. *Syntrophus aciditrophicus* sp. nov., a new anaerobic bacterium that degrades fatty acids and benzoate in syntrophic association with hydrogen-using microorganisms. Arch. Microbiol. 171, 107–114 (1999). 9914307 10.1007/s002030050685

[b28] McInerney M. J. *et al.* The genome of *Syntrophus aciditrophicus*: life at the thermodynamic limit of microbial growth. Proc. Natl. Acad. Sci. USA 104, 7600–7605 (2007). 17442750 10.1073/pnas.0610456104 PMC1863511

[b29] Stupperich E. & Konle R. Corrinoid-dependent methyl transfer reactions are involved in methanol and 3,4-dimethoxybenzoate metabolism by *Sporomusa ovata*. Appl. Environ. Microbiol. 59, 3110–3116 (1993). 16349050 10.1128/aem.59.9.3110-3116.1993 PMC182413

[b30] Kuhner C. H. *et al.* *Sporomusa silvacetica* sp, nov., an acetogenic bacterium isolated from aggregated forest soil. Int. J. Syst. Bacteriol. 47, 352–358 (1997). 9103621 10.1099/00207713-47-2-352

[b31] Stackebrandt E. *et al.* Phylogenetic analysis of the genus *Desulfotomaculum*: evidence for the misclassification of *Desulfotomaculum guttoideum* and description of *Desulfotomaculum orientis* as *Desulfosporosinus orientis* gen. nov., comb. nov. Int. J. Syst. Bacteriol. 47, 1134–1139 (1997). 9336920 10.1099/00207713-47-4-1134

[b32] Imachi H., Sekiguchi Y. & Kamagata Y. Non-sulfate-reducing, syntrophic bacteria affiliated with *Desulfotomaculum* cluster I are widely distributed in methanogenic environments. Appl. Environ. Microbiol. 72, 2080–2091 (2006). 16517657 10.1128/AEM.72.3.2080-2091.2006 PMC1393244

[b33] Juteau P. *et al.* *Cryptanaerobacter phenolicus* gen. nov., sp. nov., an anaerobe that transforms phenol into benzoate via 4-hydroxybenzoate. Int. J. Syst. Evol. Microbiol. 55, 245–250 (2005). 15653882 10.1099/ijs.0.02914-0

[b34] Qiu Y.L. *et al.* *Pelotomaculum terephthalicum* sp. nov. and *Pelotomaculum isophthalicum* sp. nov.: two anaerobic bacteria that degrade phthalate isomers in syntrophic association with hydrogenotrophic methanogens. Arch. Microbiol. 185, 172–182 (2006). 16404568 10.1007/s00203-005-0081-5

[b35] Chen C. L., Wu J. H. & Liu W. T. Identification of important microbial populations in the mesophilic and thermophilic phenol-degrading methanogenic consortia. Water Res. 42, 1963–1976 (2008). 18234274 10.1016/j.watres.2007.11.037

[b36] Kleinsteuber S. *et al.* Molecular characterization of bacterial communities mineralizing benzene under sulfate-reducing conditions. FEMS Microbiol. Ecol. 66, 143–157 (2008). 18637040 10.1111/j.1574-6941.2008.00536.x

[b37] Winderl C., Penning H., Netzer F., Meckenstock R. U. & Lueders T. DNA-SIP identifies sulfate-reducing *Clostridia* as important toluene degraders in tar-oil-contaminated aquifer sediment. ISME J. 4, 1314–1325 (2010). 20428224 10.1038/ismej.2010.54

[b38] Perkins S. D., Scalfone N. B. & Angenent L. T. Comparative 16S rRNA gene surveys of granular sludge from three upflow anaerobic bioreactors treating purified terephthalic acid (PTA) wastewater. Water Sci. Technol. 64, 1406–1412 (2011). 22179636 10.2166/wst.2011.552

[b39] Robertson W. J., Bowman J. P., Franzmann P. D. & Mee B. J. *Desulfosporosinus meridiei* sp. nov., a spore-forming sulfate-reducing bacterium isolated from gasolene-contaminated groundwater. Int. J. Syst. Evol. Microbiol. 51, 133–140 (2001). 11211250 10.1099/00207713-51-1-133

[b40] Worm P. *et al.* A genomic view on syntrophic versus non-syntrophic lifestyle in anaerobic fatty acid degrading communities. Biochim. Biophys. Acta 1837, 2004–2016 (2014). 24973598 10.1016/j.bbabio.2014.06.005

[b41] Sun W. & Cupples A. M. Diversity of five anaerobic toluene-degrading microbial communities investigated using stable isotope probing. Appl. Environ. Microbiol. 78, 972–980 (2012). 22156434 10.1128/AEM.06770-11 PMC3273032

[b42] Kuppardt A. *et al.* Phylogenetic and functional diversity within toluene-degrading, sulphate-reducing consortia enriched from a contaminated aquifer. Microb. Ecol. 68, 222–234 (2014). 24623528 10.1007/s00248-014-0403-8

[b43] Fowler S. J., Gutierrez-Zamora M. L., Manefield M. & Gieg L. M. Identification of toluene degraders in a methanogenic enrichment culture. FEMS Microbiol. Ecol. 89, 625–636 (2014). 24910080 10.1111/1574-6941.12364

[b44] Collins M. D. *et al.* The phylogeny of the genus *Clostridium*: proposal of five new genera and eleven new species combinations. Int. J. Syst. Bacteriol. 44, 812–826 (1994). 7981107 10.1099/00207713-44-4-812

[b45] Carlier J. P., Bedora-Faure M., K’ouas G., Alauzet C. & Mory F. Proposal to unify *Clostridium orbiscindens* Winter *et al.* 1991 and *Eubacterium plautii* (Séguin 1928) Hofstad and Aasjord 1982, with description of *Flavonifractor plautii* gen. nov., comb. nov., and reassignment of *Bacteroides capillosus* to *Pseudoflavonifractor capillosus* gen. nov., comb. nov. Int. J. Syst. Evol. Microbiol. 60, 585–590 (2010). 19654357 10.1099/ijs.0.016725-0

[b46] Kläring K. *et al.* *Intestinimonas butyriciproducens* gen. nov., sp. nov., a butyrate-producing bacterium from the mouse intestine. Int. J. Syst. Evol. Microbiol. 63, 4606–4612 (2013). 23918795 10.1099/ijs.0.051441-0

[b47] Schoefer L., Mohan R., Schwiertz A., Braune A. & Blaut M. Anaerobic degradation of flavonoids by *Clostridium orbiscindens*. Appl. Environ. Microbiol. 69, 5849–5854 (2003). 14532034 10.1128/AEM.69.10.5849-5854.2003 PMC201214

[b48] He Z. & Wiegel J. Purification and characterization of an oxygen-sensitive, reversible 3,4-dihydroxybenzoate decarboxylase from *Clostridium hydroxybenzoicum*. J. Bacteriol. 178, 3539–3543 (1996). 8655551 10.1128/jb.178.12.3539-3543.1996 PMC178123

[b49] Ostermann A., Gallus C. & Schink B. Decarboxylation of 2,3-dihydroxybenzoate to catechol supports growth of fermenting bacteria. Curr. Microbiol. 35, 270–273 (1997).

[b50] Ding B., Schmeling S. & Fuchs G. Anaerobic metabolism of catechol by the denitrifying bacterium *Thauera aromatica* - a result of promiscuous enzymes and regulators? J. Bacteriol. 190, 1620–1630 (2008). 18156265 10.1128/JB.01221-07 PMC2258688

[b51] Szewzyk U., Szewzyk R. & Schink B. Methanogenic degradation of hydroquinone and catechol via reductive dehydroxylation to phenol. FEMS Microbiol. Ecol. 31, 79–87 (1985).

[b52] Subramanyam R. & Mishra I. M. Biodegradation of catechol (2-hydroxy phenol) bearing wastewater in an UASB reactor. Chemosphere 69, 816–824 (2007). 17561229 10.1016/j.chemosphere.2007.04.064

[b53] Kuschk P. *et al.* Batch methanogenic fermentation experiments of wastewater from a brown coal low-temperature coke plant. J. Environ. Sci. 22, 192–197 (2010). 10.1016/s1001-0742(09)60092-9 20397405

[b54] Kato S., Yumoto I. & Kamagata Y. Isolation of acetogenic bacteria that induce biocorrosion by utilizing metallic iron as the sole electron donor. Appl. Environ. Microbiol. 81, 67–73 (2015). 25304512 10.1128/AEM.02767-14 PMC4272740

[b55] Kato S., Sasaki K., Watanabe K., Yumoto I. & Kamagata Y. Physiological and transcriptomic analyses of a thermophilic, aceticlastic methanogen *Methanosaeta thermophila* responding to ammonia stress. Microbes Environ. 29, 162–167 (2014). 24920170 10.1264/jsme2.ME14021 PMC4103522

[b56] Kato S. *et al.* The effects of elevated CO_2_ concentration on competitive interaction between aceticlastic and syntrophic methanogenesis in a model microbial consortium. Front. Microbiol. 5, 575 (2014). 25400628 10.3389/fmicb.2014.00575 PMC4214200

[b57] Kamimura N. *et al.* Characterization of the protocatechuate 4,5-cleavage pathway operon in *Comamonas* sp. strain E6 and discovery of a novel pathway gene. Appl. Environ. Microbiol. 76, 8093–8101 (2010). 20952641 10.1128/AEM.01863-10 PMC3008244

[b58] Kato S., Kai F., Nakamura R., Watanabe K. & Hashimoto K. Respiratory interactions of soil bacteria with (semi)conductive iron-oxide minerals. Environ. Microbiol. 12: 3114–3123 (2010). 20561016 10.1111/j.1462-2920.2010.02284.x

[b59] Dojka M. A., Hugenholtz P., Haack S. K. & Pace N. R. Microbial diversity in a hydrocarbon- and chlorinated-solvent-contaminated aquifer undergoing intrinsic bioremediation. Appl. Environ. Microbiol. 64, 3869–3877 (1998). 9758812 10.1128/aem.64.10.3869-3877.1998 PMC106571

[b60] Altschul S. F. *et al.* Gapped BLAST and PSI-BLAST: a new generation of protein database search programs. Nucleic Acids Res. 25, 3389–3402 (1997). 9254694 10.1093/nar/25.17.3389 PMC146917

[b61] Wang Q., Garrity G. M., Tiedje J. M. & Cole J. R. Naive Bayesian classifier for rapid assignment of rRNA sequences into the new bacterial taxonomy. Appl. Environ. Microbiol. 73, 5261–5267 (2007). 17586664 10.1128/AEM.00062-07 PMC1950982

[b62] Saitou N. & Nei M. The neighbor-joining method: a new method for reconstructing phylogenetic trees. Mol. Biol. Evol. 4, 406–425 (1987). 3447015 10.1093/oxfordjournals.molbev.a040454

[b63] Tamura K., Dudley J., Nei M. & Kumar S. MEGA4: Molecular Evolutionary Genetics Analysis (MEGA) software version 4.0. Mol. Biol. Evol. 24, 1596–1599 (2007). 17488738 10.1093/molbev/msm092

[b64] Felsenstein J. Confidence limits on phylogenies: an approach using the bootstrap. Evolution 39, 783–791 (1985). 10.1111/j.1558-5646.1985.tb00420.x 28561359

