# Supplementary material for: Methanogenic degradation of lignin-derived monoaromatic compounds by microbial enrichments from rice paddy field soil
Source: Sci Rep. 2015 Sep 24;5:14295. doi: 10.1038/srep14295 (PMC4585845; doi:10.1038/srep14295)
Supplement: Supplementary Information [file srep14295-s1.pdf]

Methanogenic complete degradation of lignin-derived monoaromatic compounds by microbial enrichments from rice paddy field soil

Souichiro Kato[1,2,3*], Kanako Chino[4], Naofumi Kamimura[4], Eiji Masai[4], Isao Yumoto[1] and Yoichi Kamagata[1,2]

[1]*Bioproduction Research Institute, National Institute of Advanced Industrial Science and Technology (AIST), 2-17-2-1 Tsukisamu-Higashi, Toyohira-ku, Sapporo, Hokkaido 062-8517, Japan*

[2]*Division of Applied Bioscience, Graduate School of Agriculture, Hokkaido University, Kita-9 Nishi-9, Kita-ku, Sapporo, Hokkaido 060-8589, Japan*

[3]*Research Center for Advanced Science and Technology, The University of Tokyo, 4-6-1 Komaba, Meguro-ku, Tokyo 153-8904, Japan*

[4]*Department of Bioengineering, Nagaoka University of Technology, 1603-1 Kamitomioka, Nagaoka, Niigata 940-2188, Japan*

*For correspondence. E-mail s.katou@aist.go.jp; Tel (+81) 11 857 8968; Fax (+81) 11 857 8915

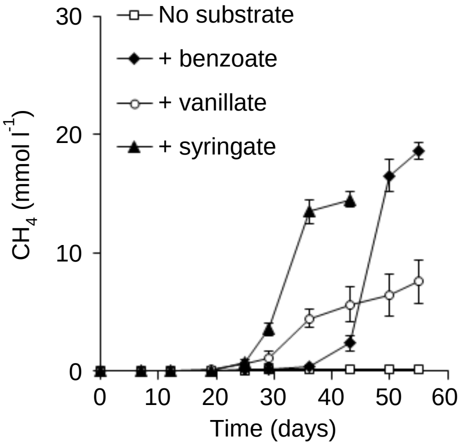

Fig. S1. Methane production by the first enrichment cultures. Production of CH_4 was monitored during the incubation of respective cultures inoculated with rice paddy field soil and supplemented with 5 mM of benzoate (closed diamonds), vanillate (open circles), or syringate (closed triangles). The data for no-amendment control (open squires) are also presented. Data are presented as the means of three independent cultures, and error bars represent standard deviations.

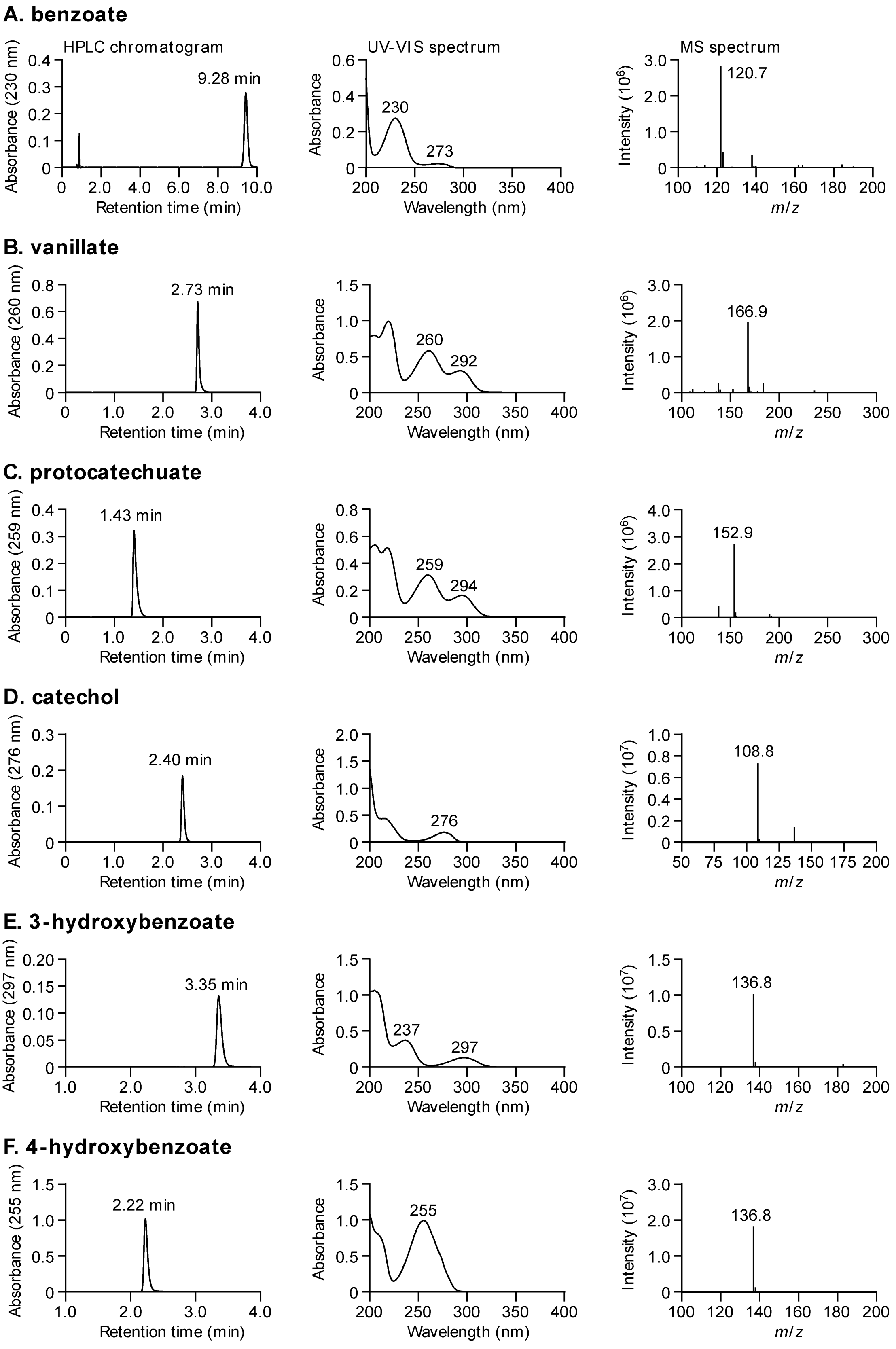
A. benzoate
HPLC chromatogram
UV-VIS spectrum
MS spectrum
Absorbance (230 nm)
9.28 min
Retention time (min)
Absorbance
230
273
Wavelength (nm)
Intensity (10^6)
120.7
m/z
B. vanillate
Absorbance (260 nm)
2.73 min
260
292
166.9
C. protocatechuate
Absorbance (259 nm)
1.43 min
259
294
152.9
D. catechol
Absorbance (276 nm)
2.40 min
276
Intensity (10^7)
108.8
E. 3-hydroxybenzoate
Absorbance (297 nm)
3.35 min
237
297
136.8
F. 4-hydroxybenzoate
Absorbance (255 nm)
2.22 min
255
136.8

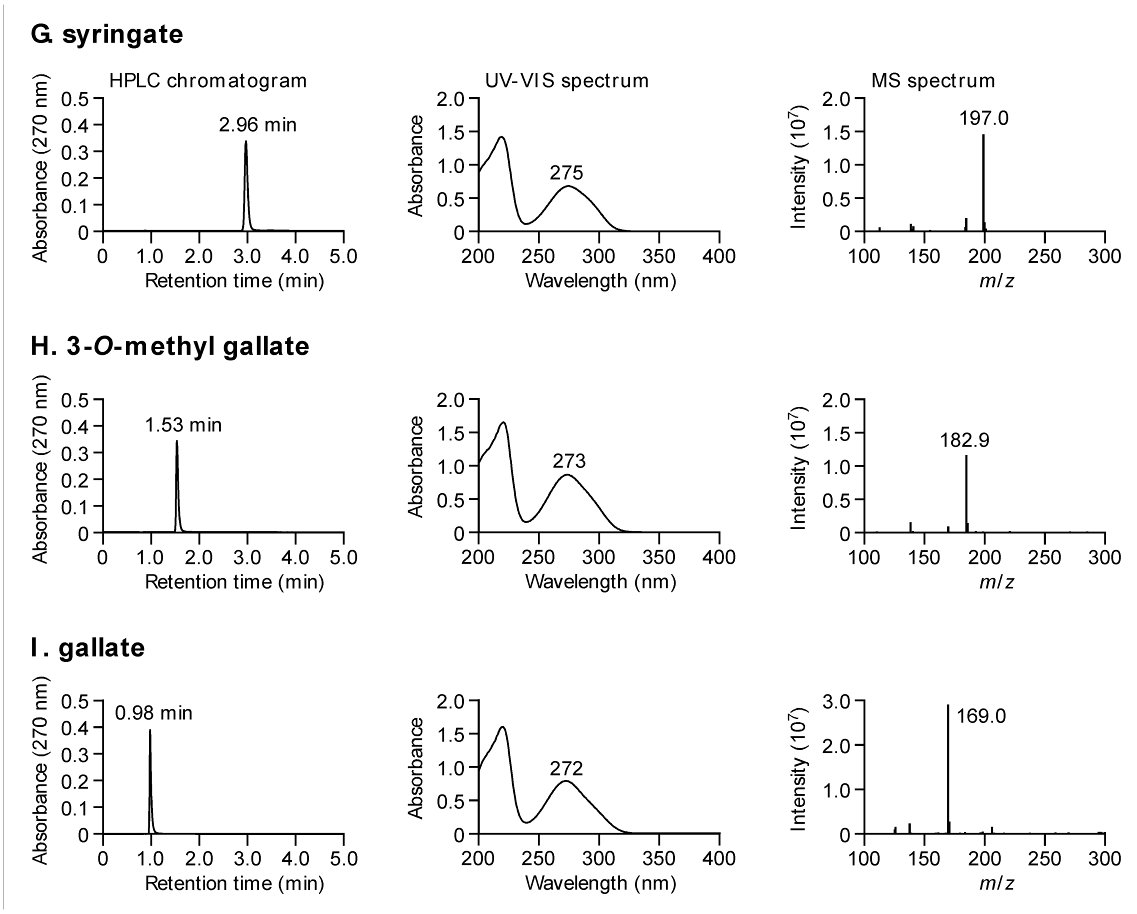

Fig. S2. LC-MS analysis of authentic benzoate (A), vanillate (B), protocatechuate (C), catechol (D), 3-hydroxybenzoate (E), 4-hydroxybenzoate (F), syringate (G), 3-*O*-methyl gallate (H), and gallate (I). HPLC chromatogram (left), UV-VIS spectra (center), and negative-ion ESI-MS spectra (right) are shown. In the HPLC analysis, compounds were detected at the following wavelength: benzoate, 230 nm; vanillate, 260 nm; PCA, 259 nm; catechol, 276 nm; 3-hydroxybenzoate, 297 nm; 4-hydroxybenzoate, 255 nm; and syringate, 3-MGA, and gallate, 270 nm. The retention times of benzoate, vanillate, PCA, catechol, 3-hydroxybenzoate, 4-hydroxybenzoate, syringate, 3-*O*-methyl gallate, and gallate were 9.28, 2.73, 1.43, 2.40, 3.35, 2.22, 2.96, 1.53, and 0.98 min, respectively.

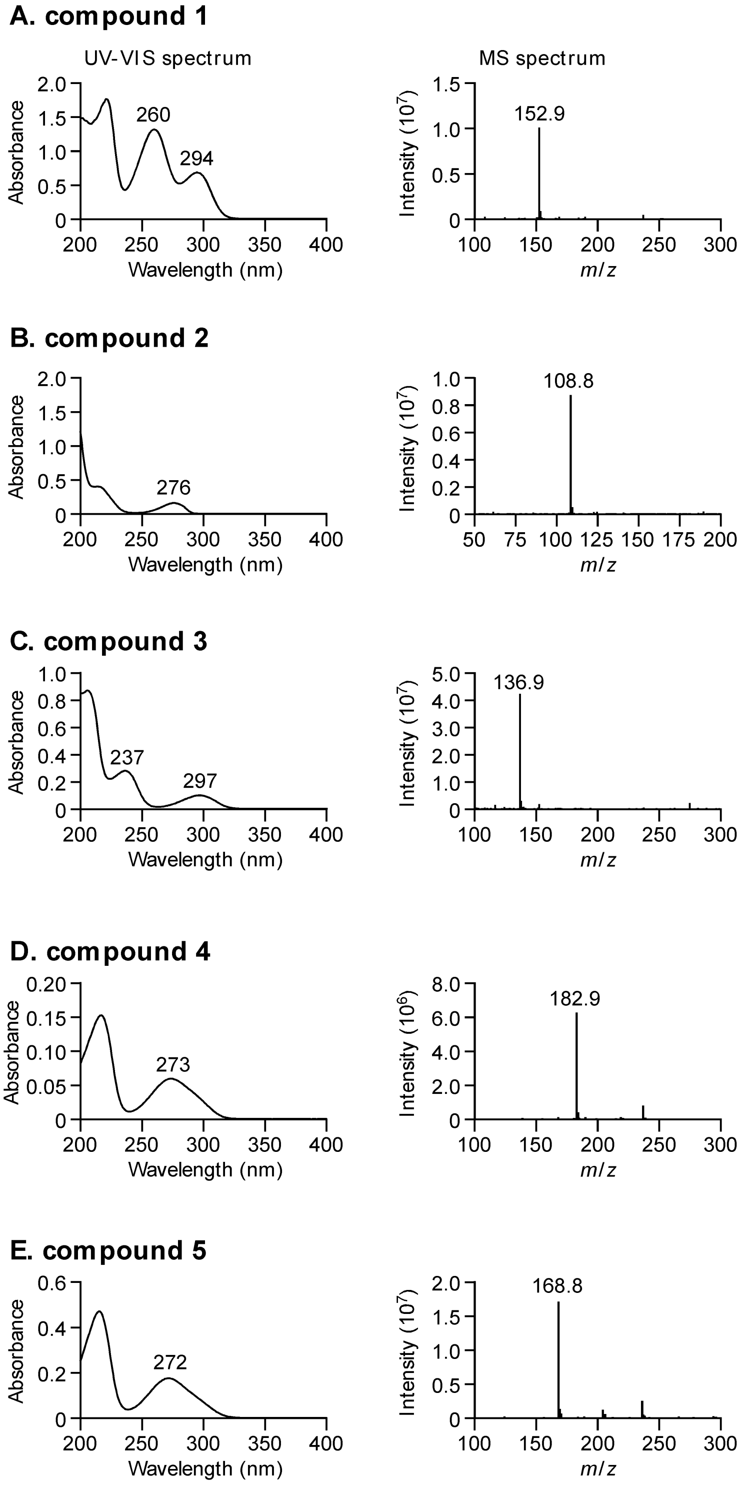

Fig. S3. UV/VIS and mass spectrum of compound I to V produced in the enrichment of vanillate and syringate. UV/VIS spectrum (left) and negative-ion ESI-MS spectrum (right) are shown.

Table S1. The archaeal phylotypes detected from the aromatics-degrading enrichment cultures.

Phylogenetic group (Family)	Phylotype	Closest relative (identity, %)	Number of clones[a]					
			BA	VA	PCA	Cat	SA	GA
Methanosarcinaceae	ArA-01	NR_104728 *Methanosarcina vacuolata* Z-761 (98.9)	6	13	18	17	18	24
Methanomicrobiaceae	ArA-02	NR_028253 *Methanoculleus palmolei* DSM 4273 (97.9)	3	8	5	6	11	3
Methanocellaceae	ArA-03	AM114193 *Methanocella arvoryzae* MRE50 (97.5)	14	3				3
Thermoplasmatales_incertae_sedis	ArA-04	CP005934 *Candidatus* “Methanomassiliicoccus intestinalis” Issoire-Mx1 (96.8)	1					
Total			24	24	23	23	29	30

[a] BA; benzoate, VA; vanillate, PCA; protocatechuate, Cat; catechol, SA; syringate, GA; gallate

Table S2. The bacterial phylotypes detected from the aromatics-degrading enrichment cultures.

Phylogenetic group		Phylo-type	Closest relative (identity, %)	Number of clones[a]					
Phylum	Family			BA	VA	PCA	Cat	SA	GA
Firmicutes	*Veillonellaceae*	ArB-01	NR_117663 *Sporomusa sphaeroides* DSM2875 (99.6)		8			22	
		ArB-02	NR_104801 *Sporomusa paucivorans* DSM3697 (89.8)					5	
		ArB-03	NR_114884 *Propionispora hippei* KS (94.1)		1				3
	Peptococcaceae	ArB-04	NR_119207 *Pelotomaculum schinkii* HH (96.8)				22		
		ArB-05	NR_025551 *Desulfosporosinus auripigmenti* OREX-4 (97.9)		10		12		
		ArB-06	NR_025757 *Cryptanaerobacter phenolicus* LR7.2 (98.4)		6	30	2		
		ArB-07	AJ229190 Unidentified eubacterium from anoxic bulk soil, clone BSV28 (97.2)				1	3	
	Ruminococcaceae	ArB-08	AB596889 *Acetivibrio* sp. 6-13 (92.0)					4	22
		ArB-09	JX101685 *Intestinimonas butyriciproducens* AP4 (97.1)		3			4	13
		ArB-10	NR_044972 *Sporobacter termitidis* SYR (96.9)				4		
		ArB-11	AF550610 *Lachnospiraceae* bacterium 19gly4 (96.8)				2	2	
		ArB-12	DQ002932 *Anaerotruncus colihominis* HKU19 (94.1)		2				
		ArB-13	NR_025025 *Papillibacter cinnamivorans* CIN1 (89.5)				1		
	Gracilibacteraceae	ArB-14	NR_115692 *Gracilibacter thermotolerans* JW/YJL-S1 (97.7)	3	2			1	
		ArB-15	NR_041236 *Lutispora thermophila* EBR46 (97.3)		1			2	
	Clostridiales_ incertae_sedis	ArB-16	NR_044093 *Proteiniborus ethanoligenes* GW (94.7)		1	1		2	
		ArB-17	X76161 *Clostridium aminobutyricum* DSM2634 (95.7)	1		1		1	1
		ArB-18	AY571338 *Sedimentibacter hongkongensis* KI (97.6)		2				

Table S2. Continued.

Phylogenetic group		Phylo-type	Closest relative (identity, %)	Number of clones[a]					
Phylum	Family			BA	VA	PCA	Cat	SA	GA
Firmicutes	*Clostridiaceae*	ArB-19	NR_112900 *Christensenella minuta* YIT12065 (90.7)		1			2	
		ArB-20	NR_026322 *Clostridium pascui* DSM10365 (99.6)			3			
		ArB-21	AB971811 *Clostridium sporogenes* JCM7849 (87.5)				1		
		ArB-22	NR_113027 *Clostridium subterminale* JCM1417 (96.7)				1		
	Erysipelotrichaceae	ArB-23	NR_028816 *Turicibacter sanguinis* MOL361 (88.4)		1				
	Syntrophomonadaceae	ArB-24	NR_115800 *Syntrophomonas wolfei* 4J5 (95.1)		1				
Proteobacteria	*Syntrophaceae*	ArB-25	NR_102776 *Syntrophus aciditrophicus* SB (99.0)	33					
	Desulfovibrionaceae	ArB-26	AJ582758 *Desulfovibrio idahonensis* CY2 (99.6)				4		
	Campylobacteraceae	ArB-27	CP007201 *Sulfurospirillum multivorans* DSM12446 (99.2)	2					
Bacteroidetes	unclassified	ArB-28	AB623230 *Bacteroidetes* bacterium 4F6B (89.8)	3	3	4	4		1
	Sunxiuqinia	ArB-29	AB921558 *Bacteroidales* bacterium Fu11-5 (91.7)				4		
	Bacteroidaceae	ArB-30	NR_125463 *Bacteroides luti* UasXn-3 (98.5)	2	1	1			
	Porphyromonadaceae	ArB-31	AB910740 *Paludibacter propionicigenes* (97.7)		1				
Ignavibacteriae	unclassified	ArB-32	NR_074698 *Ignavibacterium album* JCM16511 (85.0)		1				
Spirochaetes	*Spirochaetaceae*	ArB-33	AY695841 *Spirochaetes* bacterium SA-10 (93.2)				1		
Verrucomicrobia	unclassified	ArB-34	AY960781 Bacterium Ellin518 (97.0)				1		
Total				44	45	40	60	48	40

[a] BA; benzoate, VA; vanillate, PCA; protocatechuate, Cat; catechol, SA; syringate, GA; gallate
